# Efficacy of the Dynesys Hybrid Surgery for Patients with Multi-Segmental Lumbar Spinal Stenosis

**DOI:** 10.3389/fsurg.2022.849679

**Published:** 2022-05-26

**Authors:** Xiao Xiao, Gaoyang Chen, Song Wang, Junliang Liu, Erhu Lin, Ke Chen, Yucheng Xiang, Ke Zhan, Congcong Liu, Zhengbin Yuan, Minjie Yang, Shuyuan Zhong, Wanxin Zhen, Dazhi Yang, Songlin Peng

**Affiliations:** ^1^ Department of Spine Surgery, The First Affiliated Hospital (Shenzhen People’s Hospital), Southern University of Science and Technology, Shenzhen, China; ^2^ Department of Spine, Shenzhen Key Laboratory of Reconstruction of Structure and Function in Sports System, Shenzhen, China; ^3^ Department of Radiology, The First Affiliated Hospital (Shenzhen People’s Hospital), Southern University of Science and Technology, Shenzhen, China

**Keywords:** lumbar spinal stenosis, dynamic internal fixation, the Dynesys system, spinal fusion, hybrid surgery

## Abstract

**Objective:**

The efficacy of hybrid (Dynesys and fusion) surgery and the traditional transforaminal lumbar interbody fusion surgery was compared in patients with multi-segmental lumbar spinal stenosis.

**Methods:**

A total of 68 patients with multi-segmental lumbar spinal stenosis subjected to surgery were recruited between January 2013 and October 2020 in the First Affiliated Hospital of Southern University of Science and Technology. The patients were divided into a hybrid group (*N* = 33) and a TLIF group (*N* = 35) by surgery. After surgery, follow-up was conducted for 12 months. Between the two groups, the following parameters were compared: general conditions, clinical symptom scores, imaging parameters, and early complications.

**Results:**

A statistically significant difference in the duration of surgery was noted between the two groups. After 12 months of follow-up, the range of motion disappeared in the TLIF group, while 63.53% was preserved in the hybrid group with statistically significant differences. A statistically significant difference was identified in the Oswestry Disability Index one week after surgery. Nonetheless, no statistically significant differences were observed at the 12-month post-surgical follow-up. Pfirrmann grade showed a 3.03% upper adjacent segment degeneration rate in the hybrid group (1/33) at 12-month follow-up and 2.86% (1/35) in the TLIF group. Notably, no early complications (screw loosening and wound infection) were identified in the two groups.

**Conclusion:**

The Dynesys hybrid surgery combined the advantages of two systems of dynamic stabilization and rigid fusion. Besides, hybrid surgery is potentially a novel approach for the treatment of multi-segmental lumbar spinal stenosis.

## Introduction

Lumbar spinal stenosis is a common spinal disorder, increasing with the aging population (1–3). Conservative therapy relieves clinical symptoms in most patients during the early stages; however, surgery is essential as the disease progresses (1–4). Transforaminal lumbar interbody fusion (TLIF) surgery is preferred by spine surgeons because of its stabilization and full decompression (4, 5). Nonetheless, the potential range of motion (ROM) loss and adjacent segment degeneration (ASD) demand alternative treatments that spare the pressures and preserve the ROM (6). Among the treatments, non-fusion surgery represented by a dynamic stabilization system (Dynesys) is the most popular (7). Unlike TLIF surgery, non-fusion surgery by Dynesys preserves lumbar ROM, delaying ASD and preventing fixation failure (7–10).

Existing studies comparing Dynesys surgery with TLIF surgery are based on single-segmental lumbar spinal degeneration. Nevertheless, with the aging population and rapid lifestyle changes, the proportion of patients with multi-segmental lumbar spinal degeneration is synchronously increasing (1). For them, the degeneration of each segment may be different. Symptoms are caused by a combination of primary and secondary responsible segments in many patients, and they are usually subjected to TLIF surgery. However, this type of multi-segmental fusion potentially induces complications, including ASD and fixation failure. As such, whether alternative treatments for segments with different degeneration induce better outcomes in patients is a mystery. TLIF surgery is used for the primary responsible segment, where fusion is unavoidable. Meanwhile, Dynesys surgery is used for the secondary responsible segments, where fusion is not required, and potentially reduces the complications caused by TLIF surgery. In the past few years, spine surgeons applied this type of hybrid (Dynesys and fusion) surgery to multi-segmental degeneration patients based on this innovative idea.

Therefore, we retrospectively analysed the efficacy of the hybrid and TLIF groups in the patients with multi-segmental lumbar spinal stenosis between 2013 and 2020. This was geared toward providing a novel surgical therapy for patients with multi-segmental lumbar spinal stenosis.

## Materials and Methods

### Inclusion and Exclusion Criteria

Inclusion criteria included patients diagnosed with multi-segmental lumbar spinal stenosis (with or without lumbar disc herniation and *I*° lumbar spondylolisthesis), who underwent surgical treatment (hybrid surgery or TLIF surgery), and followed up for more than 12 months.

Exclusion criteria included patients with spinal cord injury, spinal fracture, spinal infection, spinal tumour, other spinal diseases, and chronic pain in other systems.

### Subjects

Subjects were patients admitted to the First Affiliated Hospital of SUSTC between January 2013 and October 2020, diagnosed with multi-segmental lumbar spinal stenosis, and who underwent surgical treatment. Then, the subjects were divided into a hybrid (Dynesys and TLIF) surgery group (hybrid group) and a TLIF surgery group (TLIF group) by surgery.

### Surgery

#### Hybrid surgery group

Indications of fusion segment: Severe herniation of intervertebral disc; severe spinal stenosis that requires extensive decompression, which is expected to affect the stability of the lumbar spine.
Indications of Dynesys segment: Mild and moderate disc herniation; for spinal canal stenosis, only fenestration decompression and nerve release were required, and the scope of facet joint resection was ≤50%.

Patients were operated on while under general anaesthesia in a prone position. The responsible segments were radiologically confirmed; then, the surgical segments were exposed via a midline subperiosteal muscle-stripping approach. A laminectomy was performed, followed by central canal decompression and lumbar discectomy. After preparing the endplates, the bone from the laminectomy was cleaned of residual soft tissue, milled, and then packed into a TLIF cage (Johnson & Johnson). Subsequently, the cage was inserted into the disc space of the primary responsible segment. After the pedicle screws were inserted and O-arm controlled their correct positions, the spacers were cut to accurate size and installed together with the cord. The Dynesys system was tightened with a specified preload (Zimmer). Drainage was inserted, and the fascia closed meticulously. Finally, the wound was closed.

#### TLIF surgery

Indications of fusion segment: The same as those of the above fusion segments.

Patients were operated on while under general anaesthesia in a prone position. The responsible segments were radiologically confirmed; then, the surgical segments were exposed via a midline subperiosteal muscle-stripping approach. A laminectomy was performed, followed by central canal decompression and lumbar discectomy. After preparing the endplates, the bone from the laminectomy was cleaned of residual soft tissue, milled, and then packed into TLIF cages (Johnson & Johnson). Subsequently, the cages were inserted into the disc space. After the pedicle screws were inserted, followed by the insertion of a titanium rod, and their correct positions were controlled by O-arm, drainage was inserted and the fascia was closed. Finally, the wound was meticulously closed.

All patients were allowed to rest for 3–5 days after the surgery and wore a lumbar orthosis for 3 months.

### Outcome Measures

General information includes age, gender, weight, height, BMI, surgical segments, and underlying diseases.

Before surgery, all patients underwent X-ray (anteroposterior, lateral, and dynamic), CT, and MRI examination for evaluation. One week after surgery, X-ray (anteroposterior) and CT images were obtained. The imaging evaluations 12 months after surgery included X-ray (anteroposterior, lateral, and dynamic), CT, and MRI examinations (11, 12).

The following data were collected: duration of surgery, blood loss, drainage volume, length of hospitalization, ROM of surgical segments, ROM of an upper adjacent segment, Oswestry disability index (ODI), visual analogue score (VAS), degeneration of upper adjacent segment, and early complications (including screw loosening and wound infection) (11, 12).

ROM: The ROM values of surgical segments and upper adjacent segments were measured based on a change of Cobb angle flexion/extension (13, 14).

Pfirrmann grading was used to evaluate the post-surgical degeneration rate of the upper adjacent segment.

Calculation method: The following formula was used: the number of Pfirrmann grade degeneration after surgery/the number of Pfirrmann grade before surgery × 100%. Imaging ASD was defined as Pfirrmann grade change ≥1, while symptomatic ASD was defined as the coexistence of imaging changes and corresponding segment clinical symptoms (13, 14).

### Statistical Analysis

All statistical analyses were performed using IBM SPSS 22.0. The Kolmogorov–Smirnov test (K–S test) was used to test for normal distribution. Independent *t*-tests and paired *t*-tests were used for continuous variables, while the Fisher exact test was applied for categorical data (when the frequency in the fourfold table was less than 5). The continuous variables were expressed by means ± standard deviation $\left(\overset{¯}{x}\pm s\right)$. *p* < 0.05 indicated a statistically significant difference (13, 14).

## Results

### General Information

A total of 33 cases in the hybrid group (among which 2 had hypertension, 4 had diabetes, and 1 had osteoporosis) and 35 cases in the TLIF group (among which 14 had hypertension, 8 had diabetes, and 4 had osteoporosis) were enrolled. Specifically, 22 males and 11 females were in the hybrid group, with an average age of 52.3 years, an average weight of 70.7 kg, an average height of 166.9 cm, and an average BMI of 25.1 kg/m^2^. On the other hand, 15 males and 20 females were in the TLIF group, with an average age of 66.5 years, an average weight of 66.0 kg, an average height of 161.0 cm, and an average BMI of 25.4 kg/m^2^ (Table 1). There were 11 cases of L3–L5 in the hybrid group, 16 cases of L4–S1, 5 cases of L3–S1, and 1 case of L2–S1 with 73 surgical segments and 33 upper adjacent segments. In the TLIF group, there were 25 cases of L3–L5, 9 cases of L4–S1, and 1 case of L2–S1 with 72 surgical segments and 35 upper adjacent segments (Table 2).

**Table 1:** Demographic characteristics.

	Hybrid (33)	TLIF (35)	*t*	*p*
Age, years	52.3 ± 14.1	66.5 ± 8.5	–4.994	<0.001*
M/F	15/6	9/16	–	–
Height, cm	166.9 ± 8.8	161.0 ± 8.2	2.651	0.010*
Weight, kg	70.7 ± 17.4	66.0 ± 11.4	1.276	0.207
BMI, kg/m^2^	25.1 ± 4.0	25.4 ± 3.4	–0.306	0.761

* *p < 0.05, a statistically significant difference.*

**Table 2:** Distribution of operation segments.

	Hybrid (33)	TLIF (35)
L2/3, L3/4, L4/5, L5/S1	1 (3.0%)^a^	1 (2.9%)^a^
L3/4, L4/5, L5/S1	5 (15.2%)^a^	0 (0.0%)^a^
L3/4, L4/5	11 (33.3%)^a^	25 (71.4%)^a^
L4/5, L5/S1	16 (48.5%)^a^	9 (25.7%)^a^
Surgical segments	73	72
Upper adjacent segments	33	35

*^a^* *Number (ratio).*

### Surgical Conditions

The results of surgical conditions indicated a statistical difference in the duration of surgery between the two groups. At the same time, no significant difference was noted in blood loss, drainage volume, and length of stay between them (Table 3).

**Table 3:** Surgical conditions.

	Hybrid (33)	TLIF (35)	*t*	*p*
Duration of surgery, min	246.1 ± 57.5	288.3 ± 80.6	–2.468	0.016*
Blood loss, mL	141.5 ± 31.5	160.9 ± 46.8	–1.988	0.051
Drainage volume, mL	147.2 ± 47.4	146.7 ± 44.3	0.045	0.964
Length of stay, days	18.4 ± 11.2	18.0 ± 5.6	0.200	0.842

* *p < 0.05, a statistically significant difference.*

### ROM of Surgical Segments

**Figure 1:**
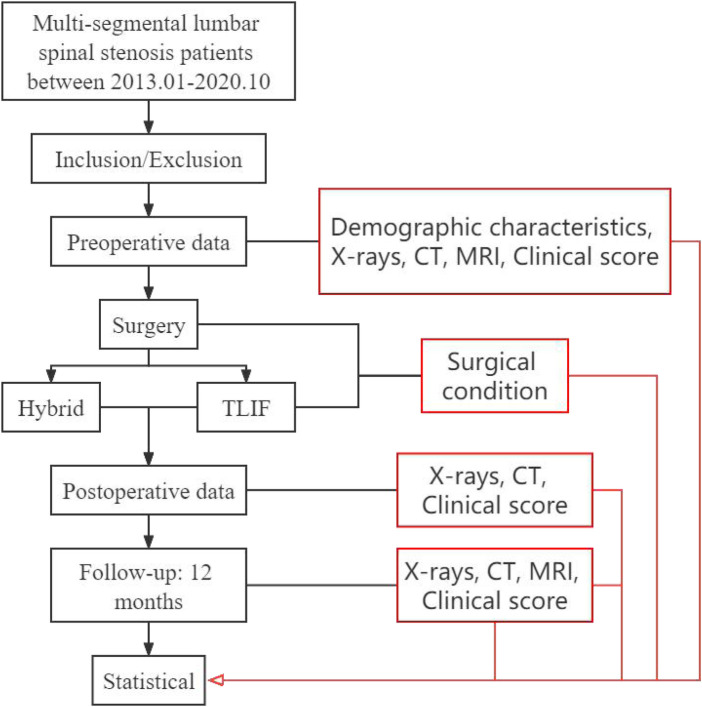
Study flow chart. The main objective of the research group was to compare the differences between hybrid surgery and TLIF surgery in the early stage of post-operation and to explore the imaging characteristics in hybrid surgery.

The ROM of the surgical segments between the two groups before surgery showed no significant statistical difference. Nevertheless, after 12 months of follow-up, the ROM of the surgical segments had disappeared in the TLIF group, while 63.53% was retained in the hybrid group; besides, the difference was statistically significant (Table 4 and Figure 1A).

**Table 4:** ROM.

	Hybrid (33)	TLIF (35)	*t*	*p*
**Surgical segments**				
Before, °	8.5 ± 4.8	7.6 ± 4.2	0.762	0.449
12-month after, °	5.4 ± 2.3	0.0 ± 0.0	5.229	0.006*
**Upper adjacent segment**			
Before, °	3.2 ± 2.0	3.7 ± 2.7	–0.904	0.370
12-month after, °	2.9 ± 2.1	3.2 ± 4.0	–0.147	0.889

* *p < 0.05, a statistically significant difference.*

### ROM of the Upper Adjacent Segment

No statistically significant difference was reported in ROM of the upper adjacent segment before and 12 months after surgery between the two groups (Table 4 and Figure 1B).

### Clinical Score

The clinical scores between the two groups indicated a statistical difference in ODI one week after surgery. Nonetheless, no statistical difference was observed at the 12-month post-surgical follow-up (Table 5 and Figure 1C). However, the VAS was not statistically different before or after surgery between the two groups (Table 5 and Figure 1D).

**Table 5:** Clinical score.

	Hybrid (33)	TLIF (35)	*t*	*p*
**ODI**				
Before	68.4 ± 10.2	70.1 ± 8.5	–0.742	0.461
1 week after	31.5 ± 3.5	33.4 ± 3.1	–2.332	0.023*
12 months after	14.1 ± 3.1	14.7 ± 2.7	–0.886	0.379
**VAS**				
Before	7.2 ± 0.9	7.5 ± 0.8	–1.03	0.307
1 week after	1.9 ± 0.6	2.3 ± 0.6	–3.083	0.003
12 months after	0.5 ± 0.5	0.6 ± 0.5	–0.46	0.647

* *p < 0.05, a statistically significant difference.*

### Post-Surgical Upper Adjacent Segmental Degeneration Rate and Early Complications

Pfirrmann’s grade demonstrated that the upper adjacent segment degeneration rate in the hybrid group was 3.03% (1/33) by 12 months post-surgery (Table 6) and 2.86% (1/35) in the TLIF group (Table 6). No significant difference was found between the two groups (Table 7). Besides, no early complications (screw loosening and wound infection) were found in the two groups.

**Table 6:** Pfirrmann grade of the hybrid group.

Pfirrmann grade	Before	12 months after				
		I	II	III	IV	V
**hybrid**					
I	–	–	–	–	–	–
II	1	–	1	–	–	–
III	24	–	–	23	1	–
IV	8	–	–	–	8	–
V	–	–	–	–	–	–
**TLIF**					
I	–	–	–	–	–	–
II	2	–	2	–	–	–
III	11	–	–	10	1	–
IV	19	–	–	–	19	–
V	3	–	–	–	–	3

**Table 7:** Degeneration rate.

		Whether degeneration		
		No	Yes	Total
Group	Hybrid	32	1	33
	TLIF	34	1	35
Total		66	2	68
*p* = 1.0				

### Typical Cases

**Figure 2:**
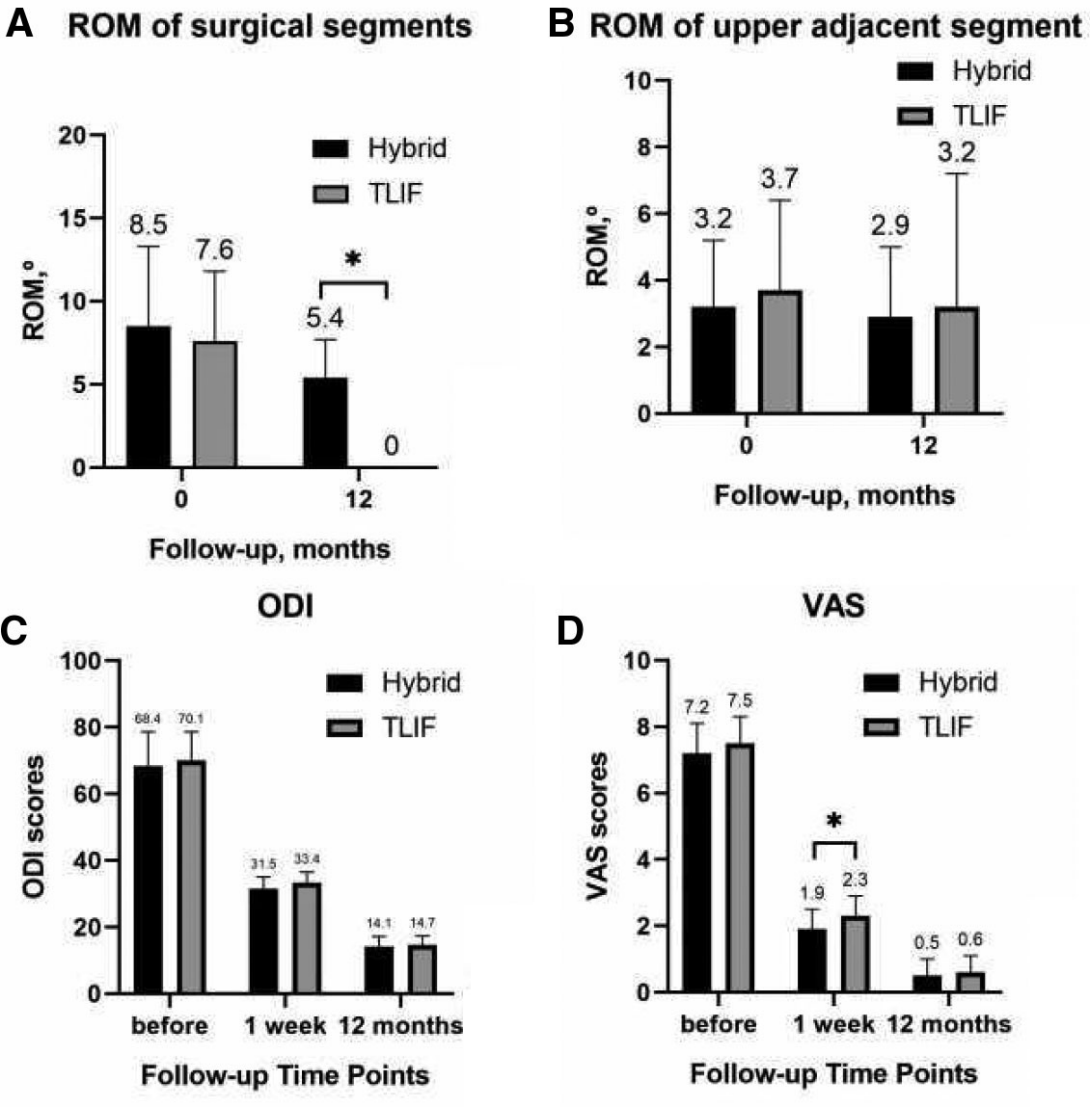
ROM of surgical segments before and 12 months after surgery. (**A**) ROM of the upper adjacent segment before and 12 months after surgery; (**B**) ODI scores; (**C**) VAS; and (**D**) for patients.

**Figure 3:**
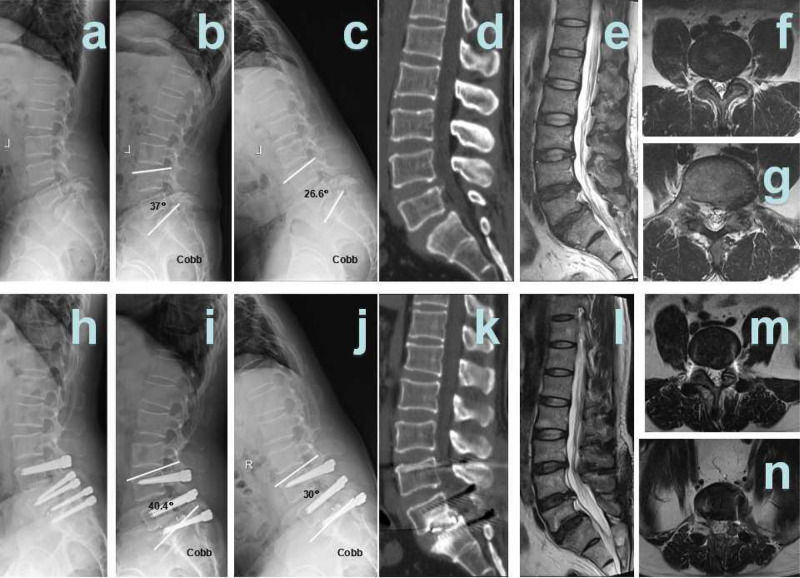
Hybrid patient 1. (**A**) Pre-surgical lateral X-ray; (**B**) pre-surgical hyper-extension X-ray; (**C**) pre-surgical hyper-flexion X-ray; (**D**) pre-surgical CT showed L4/5 disc herniation and calcification, L5 I° spondylolisthesis; (**E**) pre-surgical T2 weighted MRI sagittal plane showed L4/5, L5/S1 disc herniation; and (**F**) pre-surgical MRI showed L4/5 disc herniation and spinal stenosis; (**G**) pre-surgical MRI showed L5/S1 disc herniation and spinal stenosis; (**H**) 12 months after surgery, post-surgical lateral X-ray; (**I**) 12 months after surgery, post-surgical hyper-extension X-ray; (**J**) 12 months after surgery, post-surgical hyper-flexion X-ray; (**K**) 12 months after surgery, post-surgical CT showed interbody fusion; (**L**): 12 months after surgery, post-surgical T2-weighted MRI sagittal plane; (**M**): 12 months after surgery, post-surgical MRI sagittal plane showed that L4/5 spinal stenosis was improved significantly; and (**N**) 12 months after surgery, post-surgical MRI sagittal plane showed that L5/S1 spinal stenosis was improved significantly.

Hybrid surgery:

Patient 1 was a male, aged 39 years, diagnosed with L4/5 spinal stenosis with lumbar disc herniation and L5 spondylolisthesis. L4/5 Dynesys non-fusion hybrid L5/S1 fusion surgery was performed (Figure 2).
Patient 2 was a male, aged 48 years, diagnosed with L4/5 spinal stenosis with lumbar disc herniation and L5 spondylolisthesis. L4/5 Dynesys non-fusion hybrid L5/S1 fusion surgery was performed (Figure 3).

**Figure 4:**
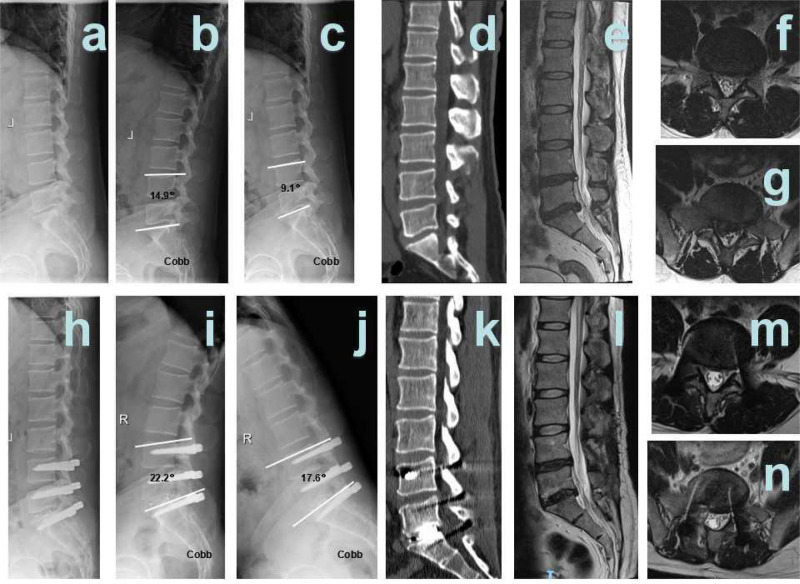
Hybrid patient 2. (**A**) Pre-surgical lateral X-ray; (**B**) pre-surgical hyper-extension X-ray; (**C**) pre-surgical hyper-flexion X-ray; (**D**) pre-surgical CT showed L5 I° spondylolisthesis; (**E**) pre-surgical T2 weighted MRI sagittal plane showed L4/5, L5/S1 disc herniation; (**F**) pre-surgical MRI showed L4/5 disc herniation and spinal stenosis; (**G**) pre-surgical MRI showed L5/S1 disc herniation and spinal stenosis; (**H**) 12 months after surgery, Post-surgical lateral X-ray; (**I**) 12 months after surgery, post-surgical hyper-extension X-ray; (**J**) 12 months after surgery, post-surgical hyper-flexion X-ray; (**K**) 12 months after surgery, post-surgical CT showed interbody fusion; (**L**) 12 months after surgery, post-surgical T2 weighted MRI sagittal plane; (**M**) 12 months after surgery, post-surgical MRI sagittal plane showed that L4/5 spinal stenosis was improved significantly; and (**N**) 12 months after surgery, post-surgical MRI sagittal plane showed that L5/S1 spinal stenosis was improved significantly.

**Figure 5:**
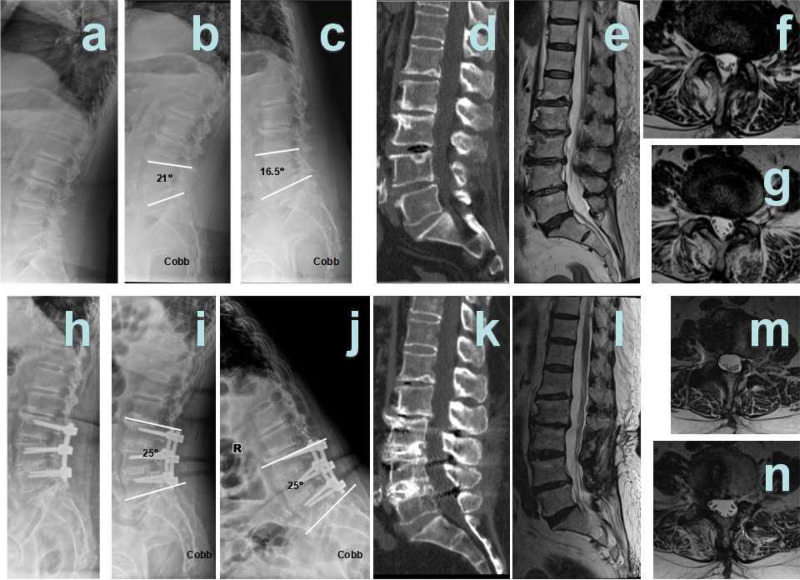
TLIF patient 1. (**A**) Pre-surgical lateral X-ray; (**B**) pre-surgical hyper-extension X-ray; (**C**) pre-surgical hyper-flexion X-ray; (**D**) pre-surgical CT showed L3/4, L4/5 spinal stenosis; (**E**) pre-surgical T2 weighted MRI sagittal plane showed L3/4, L4/5 spinal stenosis; (**F**) pre-surgical MRI showed L3/4 spinal stenosis; (**G**) pre-surgical MRI showed L4/5 spinal stenosis; (**H**) 12 months after surgery, post-surgical lateral X-ray; (**I**) 12 months after surgery, post-surgical hyper-extension X-ray; (**J**) 12 months after surgery, post-surgical hyper-flexion X-ray; (**K**) 12 months after surgery, post-surgical CT showed interbody fusion; (**L**) 12 months after surgery, post-surgical T2-weighted MRI sagittal plane; (**M**) 12 months after surgery, post-surgical MRI sagittal plane showed that L4/5 spinal stenosis was improved significantly; and (**N**) 12 months after surgery, post-surgical MRI sagittal plane showed that L5/S1 spinal stenosis was improved significantly.

TLIF surgery:

Patient 1 was a female, aged 80 years, diagnosed with L3/4, L4/5 spinal stenosis. L3/4, L4/5 TLIF surgery was performed (Figure 4).
Patient 2 was a female, aged 76 years, diagnosed with L4/5 spinal stenosis and L4 spondylolisthesis. L3/4, L4/5 TLIF surgery was performed (Figure 5).

**Figure 6:**
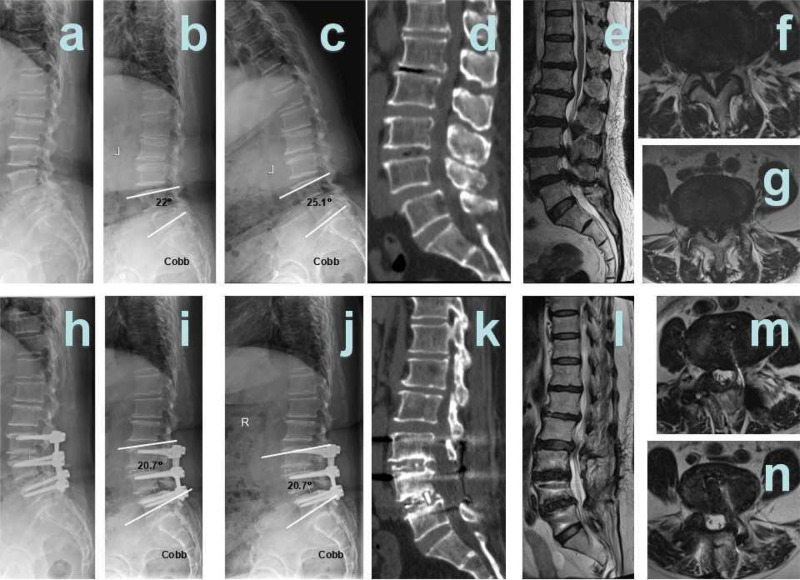
TLIF patient 2. (**A**) Pre-surgical lateral X-ray; (**B**) pre-surgical hyper-extension X-ray; (**C**) pre-surgical hyper-flexion X-ray; (**D**) pre-surgical CT showed L3/4, L4/5 spinal stenosis, L4 I° spondylolisthesis; (**E**) pre-surgical T2-weighted MRI sagittal plane showed L3/4, L4/5 spinal stenosis; (**F**) pre-surgical MRI showed L3/4 spinal stenosis; (**G**) pre-surgical MRI showed L4/5 spinal stenosis; (**H**) 12 months after surgery, post-surgical lateral X-ray; (**I**) 12 months after surgery, post-surgical hyper-extension X-ray; (**J**) 12 months after surgery, post-surgical hyper-flexion X-ray; (**K**) 12 months after surgery, post-surgical CT showed interbody fusion; (**L**) 12 months after surgery, post-surgical T2 weighted MRI sagittal plane; (**M**) 12 months after surgery, post-surgical MRI sagittal plane showed that L4/5 spinal stenosis was improved significantly; and (**N**) 12 months after surgery, post-surgical MRI sagittal plane showed that L5/S1 spinal stenosis was improved significantly.

## Discussion

### Hybrid Group Performed Better in Clinical Conditions

This study compared the clinical conditions of the two groups. The duration of surgery in the hybrid group was better than that in the TLIF group, while no significant differences were found in blood loss, drainage volume, and length of stay. As for symptoms scores, no significant differences were noted between the two groups except for ODI results at 1-week post-surgery. Although the results had a slight difference, the results remained satisfactory because of the similar clinical improvement in the two groups. This was in line with the findings of Gao et al. (15), where the Dynesys system relieved postoperative pain and improved efficacy. Therefore, the hybrid group achieved similar improvement in symptoms compared to the TLIF group and decreased surgery duration, indicating that the hybrid group performed better in clinical conditions.

### Hybrid Group Performed Better in Preserving Lumbar ROM

The prevalence of multi-segmental lumbar spinal stenosis increases with ageing. Meanwhile, the pain and intermittent neurogenic claudication caused by spinal stenosis have a severe impact on the daily life of patients and therefore necessitate surgical treatment. TLIF remains the present first-choice treatment of multi-segmental lumbar spinal stenosis. Nonetheless, to maintain the stability of the spine, TLIF surgery sacrificed the surgical segmental ROM and accelerated ASD. To address this problem, Sengupta (16) proposed a concept of “spine dynamic stabilization” to prevent ASD by preserving the ROM of surgical segments. The non-fusion surgery with the Dynesys system differed from TLIF surgery by retaining the ROM of surgical segments besides stabilizing them. This also introduced another method for treatment with multi-segmental lumbar spinal stenosis, i.e., TLIF surgery was performed for the primary responsible segment, while at the secondary responsible segment, it seemed unworthy to perform a fusion. Surgeons perform non-fusion surgery in the secondary responsible segment, i.e., treating patients with hybrid surgery. However, international studies on the Dynesys system for multi-segmental lumbar spinal stenosis primarily focused on the efficacy of the non-fusion surgery compared to TLIF surgery alone. Besides, research on hybrid surgery is still in its infancy stages.

Based on our findings, although the ROM decreased post-surgery, it retained 63.53% in the hybrid group compared to the TLIF group. This means that if the pre-operative level of motion is 8°, 5° of ROM will be preserved with the hybrid group, while TLIF group will be completely lost. This suggests that hybrid surgery is more suitable for L4–S1 because this area requires more motor function. Similarly, Zhang, Fay, and Gao et al. (7, 11, 15) found that the Dynesys system effectively stabilized the surgical segment while preserving its ROM. The cords in the Dynesys system were used to restrict lumbar flexion, while the spacer was used to restrict lumbar extension (17). Additionally, the Dynesys system maintained movement between the structures formed by the cords, spacer, and screws, making the Dynesys system retain the ROM of surgical segments in hybrid surgery (17).

### Significant Difference in Terms of ASD

The ASD mechanism is still unclear; however, Chow and Bastian et al. (18, 19) found that the increased ROM of the adjacent segment was responsible for ASD after TLIF surgery. Nevertheless, a long-term follow-up study confirming whether the Dynesys non-fusion surgery delays ASD remains unreported. Etebar et al. (20) revealed that ASD develops after 26.8 months from TLIF surgery and only 8.5–13.1 years from the Dynesys non-fusion surgery. Reports speculated that after TLIF surgery, the adjacent segment was overloaded rapidly, unable to adapt to this process, and finally caused ASD. Nonetheless, the Dynesys non-fusion surgery slowed down the process because of its structural characteristics. The adjacent segment gradually adapted to the process and slowed down degeneration.

Furthermore, Schnake et al. (21) discovered that the Dynesys non-fusion surgery exhibited a lower load in the adjacent segment than TLIF surgery, delaying the ROM increase of the adjacent segment and playing a role in preventing ASD. Pfirrmann grades of MRI signal changes in adjacent segments showed a degeneration rate in the upper adjacent segment of 3.03% (1/33) in the hybrid group and 2.86% (1/35) in the TLIF group after 12 months of follow-up. Besides, there was no significant difference between the two groups (*p* = 1.0) and no reported cases of adjacent segment degeneration. Notably, there was no statistical difference between the two groups in terms of the ROM of the adjacent segment.

### Significant Difference in Terms of Early Complications

International studies (22–24) revealed that the complication rates after Dynesys non-fusion surgery were higher than those of TLIF surgery. However, complications for both the Dynesys non-fusion surgery and TLIF surgery remain unclear due to the lack of long-term clinical studies. Our 12-month follow-up results showed no complications, including screw loosening or wound infection in the two groups; however, long-term follow-up is essential for further studies. Notably, there is a need for long-term prospective controlled studies comparing the ROM, ASD, and symptomatic clinical improvement between hybrid surgery, the Dynesys non-fusion surgery, and TLIF surgery.

### Limitation and Perspective

As a retrospective study, this article compared the efficacy of hybrid surgery and TLIF surgery in patients with multi-segmental spinal stenosis based on the Dynesys system. Kashkoush and Fay et al. (11, 12) used the Dynesys transition optima (DTO) system for hybrid surgery on patients with multi-segmental spinal stenosis, which prevented the limitations of TLIF surgery and performed the Dynesys system on the secondary responsible segment. In contrast, they used rigid fixation on the primary responsible segment. However, in our study, both primary and secondary responsible segments were treated with the Dynesys system to significantly preserve the ROM of surgical segments, which was unreported in the literature

As a limitation, this is a single-centred retrospective study with a short follow-up duration and a small sample size. A randomized controlled trial (RCT) with large samples in a multi-centre is necessary to deeply explore the efficacy of hybrid surgery for multi-segmental lumbar spinal stenosis.

From the results, it can be found that the gender, age, surgical segments, and underlying disease are different between the two groups. These factors may affect the outcome of surgery between the two groups. Considering that patients in the TLIF group are usually older and have more underlying diseases, it may increase the risk of surgical complications. Different surgical segments may also influence the results. Schilling et al.’s (25) and Cabello et al.’s (26) research on Dynesys showed that Dynesys seems to be able to disperse stress to non-adjacent segments compared with TLIF. It may have some effect on alleviating degeneration of adjacent segments. However, further research is needed. Next, subgroup analysis is necessary in order to explore the effects of age, gender, surgical segments, and underlying diseases on clinical efficacy.

Collectively, the Dynesys hybrid surgery has advantages, where it maintains vertebral stability and preserves lumbar ROM. Furthermore, the introduction of the Dynesys system in hybrid surgery reduces ASD occurrence and complications, including screw loosening or wound infection.

## Conclusion

The Dynesys hybrid surgery combined two systems of dynamic stabilization and rigid fusion. The 12-month follow-up showed that the hybrid surgery demonstrated similar symptomatic improvement, short surgery duration, and better preservation of lumbar function than the TLIF surgery. Besides, hybrid surgery is potentially a novel strategy for multi-segment lumbar spinal stenosis.

## Data Availability

The raw data supporting the conclusions of this article will be made available by the authors without undue reservation.

## References

[1] Comer C Finucane L Mercer C Greenhalgh S. SHADES of grey—the challenge of “grumbling” cauda equina symptoms in older adults with lumbar spinal stenosis. Musculoskelet Sci Pract. (2020) 45:102049. 10.1016/j.msksp.2019.102049 31439453

[2] Bydon M Alvi MA Goyal A. Degenerative lumbar spondylolisthesis: definition, natural history, conservative management, and surgical treatment. Neurosurg Clin N Am. (2019) 30(3):299–304. 10.1016/j.nec.2019.02.003 31078230

[3] Ma XL Zhao XW Ma JX Li F Wang Y Lu B Effectiveness of surgery versus conservative treatment for lumbar spinal stenosis: a system review and meta-analysis of randomized controlled trials. Int J Surg. (2017) 44:329–38. 10.1016/j.ijsu.2017.07.032 28705591

[4] Le Huec JC Seresti S Bourret S Cloche T Monteiro J Cirullo A Revision after spinal stenosis surgery. Eur Spine J. (2020) 29(Suppl 1):22–38. 10.1007/s00586-020-06314-w 31997016

[5] Ucar BY Ozcan C Polat O Aman T. Transforaminal lumbar interbody fusion for lumbar degenerative disease: patient selection and perspectives. Orthop Res Rev. (2019) 11:183–9. 10.2147/ORR.S204297 31807090 PMC6857665

[6] Wang T Ding W. Risk factors for adjacent segment degeneration after posterior lumbar fusion surgery in treatment for degenerative lumbar disorders: a meta-analysis. J Orthop Surg Res. (2020) 15(1):582. 10.1186/s13018-020-02032-7 33272288 PMC7713357

[7] Zhang Y Zhang ZC Li F Sun TS Shan JL Guan K Long-term outcome of Dynesys dynamic stabilization for lumbar spinal stenosis. Chin Med J (Engl). (2018) 131(21):2537–43. 10.4103/0366-6999.244107 30381586 PMC6213831

[8] Yu SW Yen CY Wu CH Kao FC Kao YH Tu YK. Radiographic and clinical results of posterior dynamic stabilization for the treatment of multisegment degenerative disc disease with a minimum follow-up of 3 years. Arch Orthop Trauma Surg. (2012) 132(5):583–9. 10.1007/s00402-012-1460-4 22262469

[9] Yu SW Yang SC Ma CH Kao FC Kao YH Tu YK Comparison of Dynesys posterior stabilization and posterior lumbar interbody fusion for spinal stenosis L4L5. Acta Orthop Belg. (2012) 78(2):230–9. 22696995

[10] Kuo C Huang W Wu J Tu TH Fay LY Wu CL Radiological adjacent-segment degeneration in L4–5 spondylolisthesis: comparison between dynamic stabilization and minimally invasive transforaminal lumbar interbody fusion. J Neurosurg: Spine. (2018) 29(3):250–8. 10.3171/2018.1.SPINE17993 29856306

[11] Fay L Chang C Chang H Tu TH Tsai TY Wu CL A hybrid dynamic stabilization and fusion system in multilevel lumbar spondylosis. Neurospine. (2018) 15(3):231–41. 10.14245/ns.1836108.054 30126265 PMC6226129

[12] Kashkoush A Agarwal N Paschel E Goldschmidt E Gerszten PC. Evaluation of a hybrid dynamic stabilization and fusion system in the lumbar spine: a 10 year experience. Cureus. (2016) 8(6):e637. 10.7759/cureus.637 27433416 PMC4938630

[13] Wu H Pang Q Jiang G. Medium-term effects of Dynesys dynamic stabilization versus posterior lumbar interbody fusion for treatment of multisegmental lumbar degenerative disease. J Int Med Res. (2017) 45(5):1562–73. 10.1177/0300060517708104 28661265 PMC5718723

[14] Yang B Jiang T. Comparative study of dynamic neutralization system and posterior lumbar interbody fusion in treating lumbar degenerative disease. Zhongguo Xiu Fu Chong Jian Wai Ke Za Zhi. (2013) 27(2):140–4. 23596677

[15] Gao J. Decompression and nonfusion dynamic stable system for spinal stenosis with degenerative lumbar scoliosis. Zhongguo Gu Shang. (2019) 32(10):910–3. 32512960 10.3969/j.issn.1003-0034.2019.10.007

[16] Sengupta DK. Dynamic stabilization devices in the treatment of low back pain. Neurol India. (2005) 53(4):466–74. 10.4103/0028-3886.22614 16565539

[17] Gedet P Haschtmann D Thistlethwaite PA Ferguson SJ. Comparative biomechanical investigation of a modular dynamic lumbar stabilization system and the Dynesys system. Eur Spine J. (2009) 18(10):1504–11. 10.1007/s00586-009-1077-7 19565278 PMC2899384

[18] Chow DH Luk KD Evans JH Leong JC. Effects of short anterior lumbar interbody fusion on biomechanics of neighboring unfused segments. Spine (Phila Pa 1976). (1996) 21(5):549–55. 10.1097/00007632-199603010-00004 8852308

[19] Bastian L Lange U Knop C Tusch G Blauth M. Evaluation of the mobility of adjacent segments after posterior thoracolumbar fixation: a biomechanical study. Eur Spine J. (2001) 10(4):295–300. 10.1007/s005860100278 11563614 PMC3611504

[20] Etebar S Cahill DW. Risk factors for adjacent-segment failure following lumbar fixation with rigid instrumentation for degenerative instability. J Neurosurg. (1999) 90(2 Suppl):163–9. 10.3171/spi.1999.90.2.0163 10199244

[21] Schnake KJ Schaeren S Jeanneret B. Dynamic stabilization in addition to decompression for lumbar spinal stenosis with degenerative spondylolisthesis. Spine (Phila Pa 1976). (2006) 31(4):442–9. 10.1097/01.brs.0000200092.49001.6e 16481955

[22] Kuo CH Chang PY Wu JC Chang HK Fay LY Tu TH Dynamic stabilization for L4-5 spondylolisthesis: comparison with minimally invasive transforaminal lumbar interbody fusion with more than 2 years of follow-up. Neurosurg Focus. (2016) 40(1):E3. 10.3171/2015.10.FOCUS15441 26721577

[23] Pham MH Mehta VA Patel NN Jakoi AM Hsieh PC Liu JC Complications associated with the Dynesys dynamic stabilization system: a comprehensive review of the literature. Neurosurg Focus. (2016) 40(1):E2. 10.3171/2015.10.FOCUS15432 26721576

[24] Neukamp M Roeder C Veruva SY MacDonald DW Kurtz SM Steinbeck MJ In vivo compatibility of Dynesys((R)) spinal implants: a case series of five retrieved periprosthetic tissue samples and corresponding implants. Eur Spine J. (2015) 24(5):1074–84. 10.1007/s00586-014-3705-0 25480114

[25] Schilling C Kruger S Grupp TM Duda GN Blömer W Rohlmann A. The effect of design parameters of dynamic pedicle screw systems on kinematics and load bearing: an in vitro study. Eur Spine J. (2011) 20(2):297–307. 10.1007/s00586-010-1620-6 21110209 PMC3030714

[26] Cabello J Cavanilles-Walker JM Iborra M Ubierna MT Covaro A Roca J. The protective role of dynamic stabilization on the adjacent disc to a rigid instrumented level. An in vitro biomechanical analysis. Arch Orthop Trauma Surg. (2013) 133(4):443–8. 10.1007/s00402-013-1685-x 23371399

